# In silico evaluation of WHO-endorsed molecular methods to detect drug resistant tuberculosis

**DOI:** 10.1038/s41598-022-21025-6

**Published:** 2022-10-22

**Authors:** Alice Brankin, Marva Seifert, Sophia B. Georghiou, Timothy M. Walker, Swapna Uplekar, Anita Suresh, Rebecca E. Colman

**Affiliations:** 1 Nuffield Department of Medicine, University of Oxford, Oxford, UK; 2 FIND, the Global Alliance for Diagnostics, Geneva, Switzerland; 3 Department of Medicine, University of California, San Diego, La Jolla, CA USA; 4 Oxford University Clinical Research Unit, Ho Chi Minh City, Vietnam

**Keywords:** Diseases, Infectious diseases, Tuberculosis

## Abstract

Universal drug susceptibility testing (DST) for tuberculosis is a major goal of the END TB strategy. PCR-based molecular diagnostic tests have been instrumental in increasing DST globally and several assays have now been endorsed by the World Health Organization (WHO) for use in the diagnosis of drug resistance. These endorsed assays, however, each interrogate a limited number of mutations associated with resistance, potentially limiting their sensitivity compared to sequencing-based methods. We applied an in silico method to compare the sensitivity and specificity of WHO-endorsed molecular based diagnostics to the mutation set identified by the WHO mutations catalogue using phenotypic DST as the reference. We found that, in silico, the mutation sets used by probe-based molecular diagnostic tests to identify rifampicin, isoniazid, pyrazinamide, levofloxacin, moxifloxacin, amikacin, capreomycin and kanamycin resistance produced similar sensitivities and specificities to the WHO mutation catalogue. PCR-based diagnostic tests were most sensitive for drugs where mechanisms of resistance are well established and localised to small genetic regions or a few prevalent mutations. Approaches using sequencing technologies can provide advantages for drugs where our knowledge of resistance is limited, or where complex resistance signatures exist.

## Introduction

In 2020, an estimated 10 million people became ill with tuberculosis (TB), and of these 1.5 million died from the disease^1^. *Mycobacterium tuberculosis* with antibiotic resistance poses a more severe threat; treatment success for patients with rifampicin-resistant (RR) or multidrug-resistant (MDR) TB (resistant to the most effective first-line drugs, rifampicin (RIF) and isoniazid (INH)) stands at 59% worldwide^1^. Resistance to second-line drugs used to treat MDR infections is also of concern as extensively drug-resistant (XDR) TB infections (RR/MDR plus resistance to fluoroquinolones and either bedaquiline or linezolid) can potentially develop, leaving patients with few treatment options. In order to reduce TB incidence by 80% and deaths by 90%^2^, the World Health Organisation (WHO) has identified universal drug susceptibility testing (DST) as a key component of the END TB strategy^2,3^. Universal DST is important to ensure that patients are placed on the most effective treatment regimens and to identify drug resistant strains and prevent their further spread.

Molecular diagnostic tests offer a simpler and more rapid solution to detect drug resistance in comparison to time-consuming phenotypic methods, furthermore laboratory testing is not always possible in resource limited settings due to infrastructure and trained personnel requirements^4^. *M. tuberculosis* lends itself to detection by molecular diagnostic tools because the majority of drug resistance for the most commonly used anti-TB drugs can be attributed to well characterized genetic mutations including single nucleotide polymorphisms (SNPs) and insertions or deletions^5^.

Among the several molecular diagnostic tests developed and approved by the WHO^6^ to date, GeneXpert MTB/RIF is the most widely used globally for TB and RIF resistance detection and has facilitated increased testing and RIF resistance surveillance in regions with a high TB burden^7,8^. However, individual molecular diagnostic tests are not designed to include exhaustive lists of resistance associated mutations. For example, the *rpoB* I491F mutation underlying an MDR outbreak in Eswatini was not detected by GeneXpert MTB/RIF^9^ which may have contributed to spread of undetected RIF resistance in South Africa^10^. Current molecular diagnostic tests can also give false positive resistance diagnosis, which could result in the unnecessary, longer treatment regimens including more toxic antitubercular drugs. For instance, a mutation that does not confer resistance, *gyrA* A90G, prevents hybridization of a wild-type probe in the Hain Genotype MTBDRsl v1 and v2 assays resulting in a misleading test interpretation of fluoroquinolone resistance^11,12^.

Whole genome sequencing (WGS) offers an alternative to current molecular diagnostic testing; it provides a comprehensive report of the genetic variation present in a *M. tuberculosis* isolate and can be used to successfully predict resistance and susceptibility to antibiotics in both TB^13–15^ and other infectious diseases^16,17^. This method of detecting SNPs associated with resistance is already being widely used for comprehensive DST in some high-income countries, including the United Kingdom where all *M. tuberculosis* isolates now undergo WGS. However, the current WGS workflow still has time and infrastructure limitations as *M. tuberculosis* bacilli must be isolated and cultured from the clinical sample prior to sequencing to ensure sufficient coverage of the bacterial genome^18^. Targeted next generation sequencing (tNGS) could be a more appropriate solution for resource limited settings as it can be implemented directly from clinical samples, offering a more rapid resistance diagnosis^19^, and performs comparably to WGS platforms for detecting resistance conferring mutations^20–22^.

Ultimately, the success of both sequencing and molecular diagnostic tests for TB resistance prediction relies on the production of a comprehensive catalogue of TB genetic mutations. This need has led to the collection of large, global, matched genotypic and phenotypic datasets, from which statistical tests, machine learning or genome wide association studies can be used to identify the mutations that are associated and not associated with resistance^23^. Most recently, the WHO published a catalogue of over 17,000 mutations associated with resistance to 13 anti-TB drugs using data from over 38,000 *M. tuberculosis* isolates using a standardized statistical approach^15,24^. The catalogue is therefore recommended for the standardized interpretation of sequencing based DST approaches and the mutations identified can also be used to inform the development of novel molecular diagnostic tests.

Although next generation sequencing (NGS) approaches offer an attractive solution to some of the problems encountered when using molecular diagnostic tests, sequencing approaches are not yet implementable in all settings due to the infrastructure requirements. It is therefore important to evaluate how well different molecular tests could detect resistance by the mutations that they cover, and how the performance compares to catalogue-based resistance prediction. An in silico evaluation method has particular advantages as it can quickly evaluate proposed lists of mutations for identifying resistance in new or revised drug resistance assays in future. In this study, we measure the in silico performance of WHO endorsed molecular assays, based solely on the mutations they cover, in predicting drug resistance within the globally diverse dataset used to derive the WHO catalogue, comparing it to the performance of the mutations from the WHO catalogue itself, using phenotypic DST as the reference standard.

## Methods

### Compilation of mutations

For our exhaustive list of resistance associated mutations, we used the WHO 2021 catalogue and included mutations that were categorised as (1) associated with resistance or (2) associated with resistance interim^15,24^. We evaluated WHO endorsed molecular diagnostic tests that detected resistance to one or more of the following drugs: rifampicin, isoniazid, pyrazinamide, ethionamide, levofloxacin, moxifloxacin, amikacin, capreomycin or kanamycin. Where documented, specific mutations detected by mutant probes and regions where any mutation would result in disruption of binding of a wild-type probe were identified based on package inserts or literature (Table 1). Specific probes used for detecting mutations not associated with resistance, or positions in regions that wild type probes are agnostic to were also included where available. If a list of specific probes or regions was not available for any molecular diagnostic assay, secondary evidence from literature was used. The list of mutations detected by each diagnostic test for use in in silico evaluations of the tests is summarised in Table 1.

**Table 1:** Mutations detected by WHO-endorsed molecular diagnostic tests.

Test	Drug	Resistant mutant probes	Wild type probes	Susceptible probes/agnostic	References
Abbott RealTime MTB RIF/INH	Rifampicin		*rpoB* **426:452**		^6,25,26^
	Isoniazid	*katG* **S315T** *inhA* c-15t	*katG* **315** *inhA* -15		^6,25,26^
BD MAX MDR-TB	Rifampicin		*rpoB* **426:452**		^6,27^
	Isoniazid		*katG* **315** *inhA* -15		
Roche cobas MTB-RIF/INH	Rifampicin	*rpoB* L430P, Q432K, Q432L, Q432P, D435G, D435V, D435Y, S441L, S441Q, S441W, H445D, H445L, H445N, H445R, H445Y, S450L, S450W, L452P			^6,28^
	Isoniazid	*katG* **S315I, S315N, S315T** *inhA* t-8a, t-8c, c-15t			^6,28^
Hain FluoroType MTBDR VER 2.0	Rifampicin	*rpoB* T427A, S428T, E429H, L430P**, S431K, Q432L, Q432P, Q432R, D435A, D435F, D435V, D435Y, N437I, S441L, S441Q, H445C, H445D, H445G, H445L, H445N, H445P, H445Q, H445R, H445S, H445Y, R448K, S450F, S450L, S450Q, S450W, L452E, L452P**			^6,29^
	Isoniazid	*katG* **S315T**, **S315N**, **S315R**, *inhA* t-8a, t-8c, t-8 g, g-9a, c-15t, a-16 g, g-17t			^6,29^
Nipro Genoscholar PZA-TB II	Pyrazinamide		*pncA* -17:**185**	*pncA* **G60G**, **S65S**, **T142T**	^6,30,31^
Nipro Genoscholar NTM + MDRTB detection kit 2	Rifampicin	*rpoB* **D435V, H445Y, H445D, S450L**	*rpoB* 428:453		^6,31^
	Isoniazid	*katG* **315 T, 315 N** *inhA* a-16 g, c-15t, t-8c, t-8a	*katG* **294:299, 313:318, 323:330** *inhA* -17:-3, **6:11**		^6,31^
Hain GenoType MTBDRplus Ver 2.0	Rifampicin	*rpoB* **D435V, H445Y, H445D, S450L**	*rpoB* **424:452**		^6,32^
	Isoniazid	*katG* **315 T, 315 N** *inhA* a-16 g, c-15t, t-8c, t-8a	*katG* **315** *inhA* -16:-8		^6,32^
Hain GenoType MTBDRsl Ver 1.0	Fluoroquinolones	*gyrA* **A90V, S91P, D94A, D94N/Y, D94G, D94H**	*gyrA* **85:96**	*gyrA* **S95T**	^6,11,29,33^
	Aminoglycosides	*rrs* a1401g, g1484t	*rrs* 1401:1402, 1484		^6,29,33^
Hain GenoType MTBDRsl Ver 2.0	Fluoroquinolones	*gyrA* **A90V, S91P, D94A, D94N/Y, D94G, D94H** ***gyrB* N499D, E501V**	*gyrA* **85:96** *gyrB* **497:502**	*gyrA* **S95T**	^6,11,32^
	Kanamycin	*rrs* a1401g, g1484t *eis* c-14t	*rrs* 1401:1402, 1484 *eis* -37, -15:-10		^6,32,34^
	Amikacin, Capreomycin	*rrs* a1401g, g1484t *eis* c-14t	*rrs* 1401:1402, 1484		^6,32,34^
INNO-LiPA Rif.TB	Rifampicin	*rpoB* **D435V, H445Y, H445D, S450L**	*rpoB* **428:453**		^35,36^
TrueNat MTB/Rif	Rifampicin	*rpoB* **Q432L, Q432P, D435G, D435V, D435Y, H445D, H445L, H445N, H445R, H445Y, S450L, S450W, L452P**			^6,37,38^
Cepheid Xpert MTB/RIF	Rifampicin		*rpoB* **426:452**		^6,39^
Cepheid Xpert MTB/RIF Ultra	Rifampicin		*rpoB* **426:452**	*rpoB* **Q513Q, F514F**	^6,40^
Cepheid Xpert MTB/XDR	Isoniazid	*katG* **S315T** *inhA* c-15t, t-8a, t-8c, *fabG1* **L203L** (g609a) *ahpC* g-48a, c-39t, g-6a			^6,41–43^
	Ethionamide	*inhA* c-15t, t-8a, t-8c			^6,41–43^
	Fluoroquinolones	*gyrA* **G88A, G88C, A90V, S91P, D94A, D94G, D94H, D94N, D94Y** *gyrB* **D461N, D461V, N499T, E501D, E501V**			^6,41–43^
	Aminoglycosides	*rrs* a1401g, c1402t *eis* g-37t, c-14t, c-12t, g-10a, c-8del			^6,41–43^

Regions are denoted by ‘:’ and are inclusive. Positions relating to codons rather than nucleotide positions are in bold. Amino acids are denoted by upper case letters and nucleotides by lower case. Promoter mutations are denoted by a ‘–’. The *eis* c-14t probe is included for aminoglycoside resistance detection by GenoType MTBDR*sl* v2 as the mutation is included in the WHO 2021 catalogue as associated with resistance to these drugs.

### Dataset and filtering

A total of 38,126 isolates used to compile the 2021 WHO catalogue of *M. tuberculosis* complex mutations associated with drug resistance with phenotypic and WGS data for either rifampicin, isoniazid, pyrazinamide, ethionamide, levofloxacin, moxifloxacin, amikacin, capreomycin or kanamycin were considered for this analysis^15,24^. Isolates with known resistance conferring mutations identified via sequencing and a susceptible phenotype for the corresponding drug were not removed from this sample set; although these samples may have been mislabelled, they were kept in the dataset to reflect real world performance, where there will be a degree of mislabelling and phenotypic error. The WGS data that we used was processed for the compilation of the 2021 WHO catalogue, and as such, nucleotide positions where the bioinformatic pipeline used reported no data or where there was equal support for two different nucleotides (null call) and positions where there was evidence for multiple nucleotides or where there was insufficient coverage (filter-fail) were assumed wild-type. Therefore, heterozygous calls (fraction of read support < 90%) were not considered for this analysis, although we note that these could be picked up by WGS and catalogue-based prediction pipelines, as well as some molecular diagnostic tests^44^.

For this analysis we excluded phenotypic data for drugs where one or more of the corresponding genes interrogated by either the catalogue or a molecular test (Table S1) had an excessive number of filter-fail or null calls for the variants identified, on the assumption that the genetic data were unreliable. The definition used for ‘excessive’ was the probability of that many filter fail or null calls being less than 1%, assuming a Poisson distribution^15,24^. Upon application of these filtering criteria, 38,111 isolates remained for further analysis; a breakdown of the number of isolates with phenotypic and genetic information for each drug is presented in Table 2. The origin of the 38,111 samples in the final set is shown in Table S2.

**Table 2:** Number of isolates with both WGS sequencing and phenotypic data for each drug.

Drug	Total isolates	Phenotpyic resistant isolates	Percentage phenotypic resistant isolates
Rifampicin	34,416	9869	28.7
Isoniazid	34,440	12,195	35.4
Pyrazinamide	15,934	2337	14.7
Ethionamide	14,009	2978	21.3
Levofloxacin	18,386	3125	17.0
Moxifloxacin	13,440	1880	14.0
Amikacin	17,093	1295	7.6
Capreomycin	11,622	974	8.4
Kanamycin	16,277	1488	9.1

The total number of isolates does not sum to 38,111 because several isolates have phenotypic DST for multiple drugs.

### In silico performance of sequencing and molecular diagnostic tests

To evaluate the in silico sensitivity and specificity of sequencing compared to phenotypic DST as reference, we examined the proportion of phenotypically resistant and susceptible isolates in the dataset described that contained mutations from the 2021 WHO catalogue that were ‘associated with resistance’ or ‘associated with resistance—interim’. We acknowledge that the in silico performance of the catalogue may be overestimated as the same isolates used to build the catalogue were used for this evaluation, however no other comparably large or diverse dataset was available to use for the phenotypic comparisons.

In silico evaluations of the individual molecular diagnostic tests were performed using the mutations listed in Table 1. For diagnostic tests where a genetic region is interrogated by a wild type probe, any mutation within that region was considered detected as resistant by that test, and therefore called resistant for this analysis, unless the mutation was one where the probe is specifically designed to be agnostic, for example *gyrA* S95T (Table 1). Similarly, if a mutation within the region was identified as a non-resistance conferring mutation by a specific probe (e.g. *pncA* S65S, Table 1), the isolate was called susceptible.

Several of the tests identify both specific mutations and, more generally, simply the presence of any mutation within broader genetic regions, and they therefore are interpreted differently i.e. ‘resistance detected’ (where a specific mutation is detected by a mutant probe) or ‘resistance inferred’ (where the test can infer resistance by detecting any mutation within a given region covered by wild type probes). We therefore performed sensitivity and specificity analyses separately for the two different possible interpretations in these cases, although we acknowledge the likelihood that only one interpretation would be used in the clinic.

**Figure 1:**
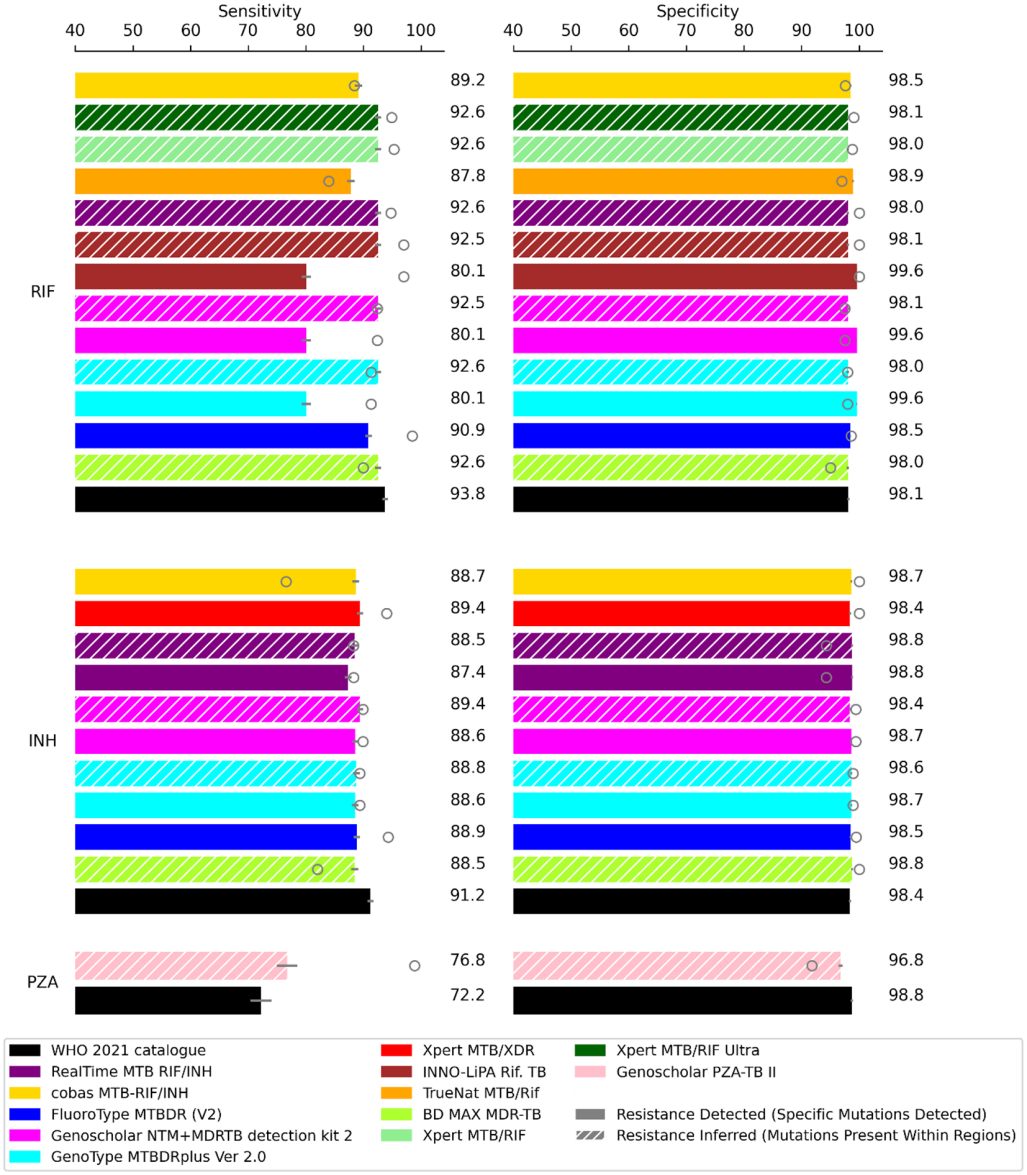
Sensitivity and specificity of WHO catalogue mutations and mutations detected by molecular diagnostic tests for predicting resistance to first-line antitubercular drugs. Error bars show 95% confidence intervals and point plots indicate performance of the molecular diagnostic test in clinical trials, these values are listed in Table 3. A full table of results is presented in Table S3. Drug acronyms: *RIF* rifampicin, *INH* isoniazid, *PZA* pyrazinamide.

**Figure 2:**
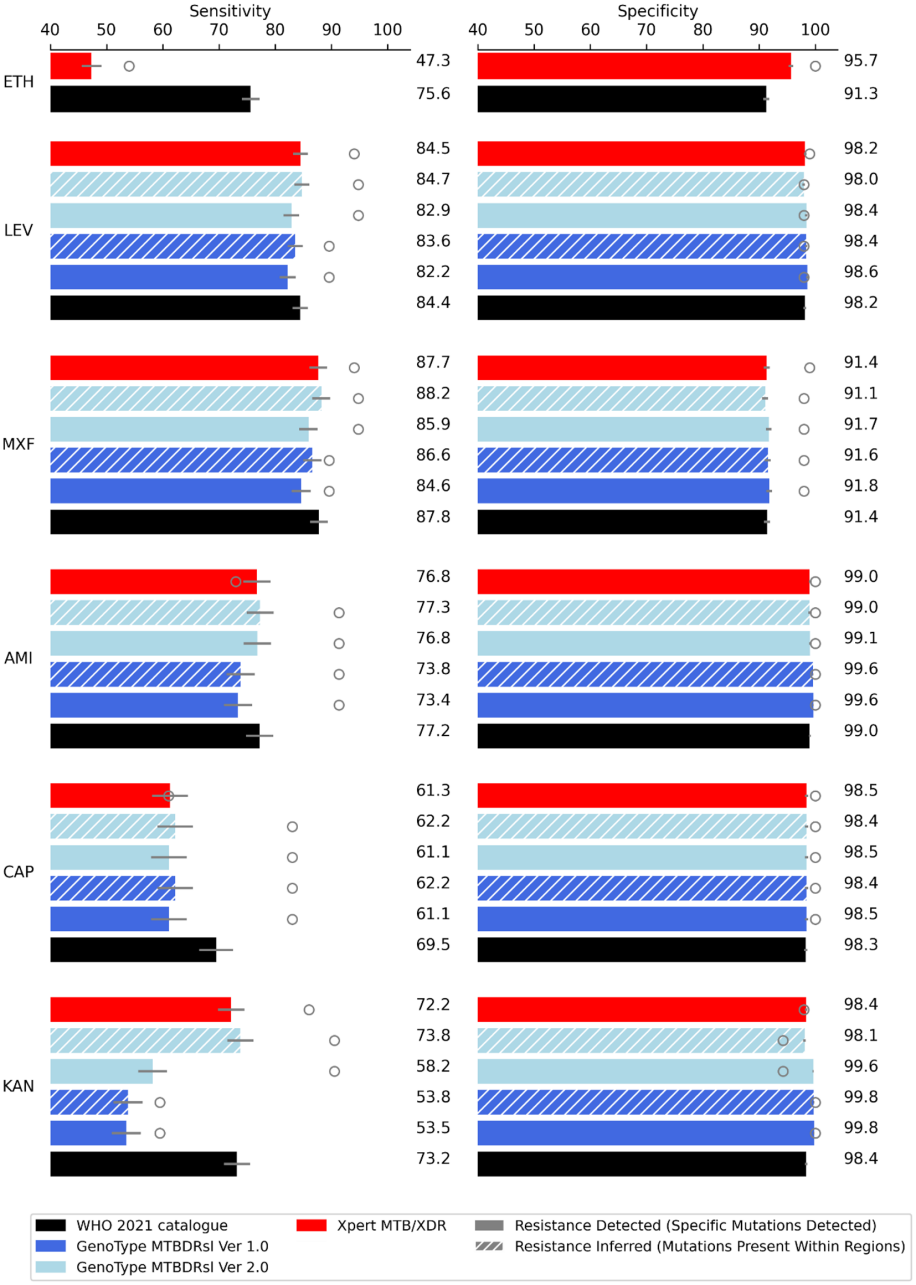
Sensitivity and specificity of WHO catalogue mutations and mutations detected by molecular diagnostic tests for predicting resistance to antitubercular drugs not used in first-line treatment. Error bars show 95% confidence intervals and point plots indicate performance of molecular diagnostic tests in clinical trials, these values are listed in Table 3. A full table of results is presented in Table S3. Drug acronyms: *ETH* ethionamide, *LEV* levofloxacin, *MXF* moxifloxacin, *AMI* amikacin, *CAP* capreomycin, *KAN* kanamycin.

All the analyses were performed and figures prepared using python3 notebooks and python3 packages. The Wilson Score method was used to calculate 95% confidence intervals for individual sensitivity and specificity calculations^45^. The files and notebooks necessary to run the in silico analysis presented in this manuscript, and reproduce Figs. 1 and 2, can be accessed at https://github.com/alicebrankin/In-silico-evaluation-of-WHO-endorsed-molecular-methods-to-detect-drug-resistant-tuberculosis.

### Compilation of clinical performance of molecular diagnostic tests

Where possible, we used pooled clinical sensitivity and specificity data from systematic reviews of tests to indicate the likely performance of the molecular diagnostic tests for in silico analyses. Where this information was not available, we used clinical tests with the largest possible and most geographically diverse sample set. Reported sensitivities and specificities from literature were compared to traditional phenotypic DST as standard^26,46–55^, except for Xpert MTB/XDR where a composite reference standard comprising phenotypic DST and WGS data was used^56^. The clinical sensitivity and specificity for each of the tests analysed in this study is shown in Table 3.

**Table 3:** Clinical performance of molecular diagnostic tests by drug.

Test	Drug	Sensitivity (95% CI)	Specificity (95% CI)	Refs.
Abbott RealTi*m*e RIF/INH	Isoniazid	88.3% (80.0 to 94.0)	94.3% (86.6 to 97.7)	^26^
	Rifampicin	94.8% (83.3 to 98.3)	100% (97.0 to 100)	^26^
BD MAX MDR-TB	Isoniazid	82% (63 to 92)	100% (98–100%)	^46^
	Rifampicin	90% (60 to 98)	95% (91 to 97)	^46^
Hain FluoroType MTBDR VER 2.0	Isoniazid	94.3% (85.3 to 98.2)	99.5% (96.8 to 100)	^47^
	Rifampicin	98.5% (90.6 to 100)	98.6% (95.7 to 99.6)	^47^
Hain GenoType MTBDR*sl* v1.0	Amikacin	91.3% (79.7 to 96.6)	100% (95.5 to 100)	^48^
	Capreomycin	83.0% (70.8 to 90.8)	100% (95.1 to 100)	^48^
	Fluoroquinolones	89.6% (80.8 to 94.6)	98% (89.5 to 99.7)	^48^
	Kanamycin	59.5(48.1 to 69.9)	100% (93.2 to 100)	^48^
Hain GenoType MTBDR*sl* v2.0	Amikacin	91.3% (79.7 to 96.6)	100% (95.5 to 100)	^48^
	Capreomycin	83.0% (70.8 to 90.8)	100% (95.1 to 100)	^48^
	Fluoroquinolones	94.8% (87.4 to 98.0)	98% (89.5 to 99.7)	^48^
	Kanamycin	90.5% (81.7 to 95.3)	94.3% (84.6 to 98.1)	^48^
Hain GenoType MTBDR*plus* V1	Isoniazid	84.3% (76.6 to 89.8)	99.5% (97.5 to 99.9)	^49^
	Rifampicin	98.1% (95.9 to 99.1)	98.7% (97.3 to 99.4)	^49^
Hain GenoType MTBDR*plus* V2	Isoniazid	89.4% (84.3 to 93.3)	98.9% (96.0 to 99.9)	^50^
	Rifampicin	91.3% (86.0 to 95.0)	98.0% (95.0 to 99.5)	^50^
INNO-LiPA Rif.TB	Rifampicin	96.9%	100.0%	^51^
Nipro NTM + MDRTB	Isoniazid	89.9% (84.9 to 93.8)	99.4% (96.9 to 100.0)	^50^
	Rifampicin	92.4% (87.4 to 95.9)	97.5% (94.3 to 99.2)	^50^
Nipro Genoscholar PZA-TB II	Pyrazinamide	98.9% (97.5 to 100)	91.8% (87.9 to 95.8)	^52^
Roche cobas MTB-RIF/INH	Isoniazid	76.6% (62.8 to 86.4)	100.0% (90.8 to 100.0)	^53^
	Rifampicin	88.4% (75.5 to 94.9)	97.6% (87.4 to 99.6)	^53^
Truenat MTB-RIF	Rifampicin	83% (70 to 92)	97% (93 to 98)	^54^
Cepheid Xpert MTB/RIF	Rifampicin	95.3% (90.0 to 98.1)	98.8% (97.2 to 99.6	^55^
Cepheid Xpert MTB/XDR	Amikacin	73% (62 to 81)	100% (98 to 100)	^56^
	Capreomycin	61% (49 to 70)	100% (98 to 100)	^56^
	Ethionamide	54% (50 to 61)	100% (97 to 100)	^56^
	Fluoroquinolones	94% (90 to 96)	99% (97 to 100)	^56^
	Isoniazid	94% (92 to 96)	100% (94 to 100)	^56^
	Kanamycin	86% (81 to 91)	98% (96 to 99)	^56^
Cepheid Xpert MTB/RIF Ultra	Rifampicin	94.9% (88.9 to 97.9)	99.1% (97.7 to 99.8)	^55^

Where possible, sensitivities and specificities are pooled from multiple clinical studies in systematic reviews. Please see references for the methods used to determine the clinical sensitivity and specificity of tests.

## Results

To examine the performance of the WHO catalogue of mutations and the mutations identified by molecular diagnostic tests compared to phenotypic DST, we constructed in silico versions of molecular diagnostic tests based on lists of mutations that they detect (Table 1). We examined all drugs for which a WHO endorsed molecular test is available, including first-line agents (rifampicin, isoniazid and pyrazinamide) fluoroquinolones (levofloxacin and moxifloxacin), injectable drugs (amikacin, capreomycin and kanamycin), and ethionamide.

### Rifampicin

The WHO catalogue of mutations, which includes mutations outside of the rifampicin resistance determining region (RRDR) of *rpoB*, identified 93.8% of the phenotypically rifampicin-resistant isolates in the dataset (Fig. 1). This was higher than any of the other molecular tests (80.1–92.6%), which do not detect mutations outside of the *rpoB* RRDR.

Several molecular diagnostics can infer rifampicin resistance based upon detection of any mutation within the RRDR, but the tests differ in the exact genetic positions they target (Table 1). Despite these differences, there was no notable difference in sensitivity or specificity for the sets of mutations detected by Xpert MTB/RIF, Xpert MTB/RIF Ultra, Genoscholar NTM + MDRTB detection kit v2, GenoType MTBDR*plus* ver 2.0, INNO LiPA MTB Rif, BD MAX MDRTB and Abbott Realti*m*e MTB RIF/INH (Fig. 1). The in silico specificity of all molecular tests and the catalogue was high, at over 98%, and tests where specific mutations are detected were more specific than any resistance inferred test interpretation and the catalogue (Fig. 1). Notably, the Xpert MTB/RIF Ultra, which includes specific probes to exclude non-resistance conferring mutations in the RRDR to reduce false positive resistance predictions, did not significantly outperform GeneXpert MTB/RIF in terms of in silico specificity on this dataset. For tests where both resistance detected and resistance inferred interpretations are possible (Genoscholar NTM + MDRTB detection kit v2, GenoType MTBDR*plus* V2, INNO LiPA MTB Rif), the sensitivity was much lower at 80.1%, however the tests in question only detect four specific mutations. The in silico results showed that tests that identify a greater number of mutations to detect resistance performed better in terms of sensitivity (FluoroType MTBDR VER 2.0, cobas MTB RIF/INH and Truenat MTB/RIF achieved 90.9%, 89.2% and 87.8% sensitivity, respectively), though the mutations identified by these tests still had lower sensitivity than resistance-inferred interpretations.

### Isoniazid

Similarly to rifampicin resistance, the catalogue mutations identified more isoniazid-resistant isolates in the dataset in the in silico analysis than any of the molecular diagnostic tests, with 91.2% sensitivity (Fig. 1). The specificity of the catalogue mutations was not significantly different from the molecular tests, except for resistance-detected and resistance inferred interpretations of RealTi*m*e MTB RIF/INH and for BD MAX MDRTB which had 0.37%, 0.35% and 0.35% higher specificity respectively. There was no significant difference in sensitivity between any of the in silico results for the molecular diagnostic tests, despite the differences in mutations identified and genes probed, except for the resistance-detected based interpretation of RealTi*m*e MTB RIF/INH, which had lower sensitivity. Although there was no significant difference between in silico sensitivity compared to other molecular diagnostic tests, the Xpert MTB/XDR test identified the most phenotypically resistant isolates, with 89.4% sensitivity. However, it is notable that the in silico sensitivity of Xpert MTB/XDR for isoniazid resistance detection was lower than that seen clinically.

### Pyrazinamide

For pyrazinamide, the mutations identified by Genoscholar PZA-TB II (which includes any mutation in *pncA* and up to 17 base pairs upstream) allowed a significantly higher proportion of pyrazinamide resistance to be identified in silico than the catalogue mutations, with 76.8% versus 72.2% sensitivity respectively (Fig. 1). However, the in silico sensitivity of Genoscholar PZA-TB II compared to phenotypic DST was much lower than the sensitivity seen in clinical evaluation. Despite inclusion of probes to identify commonly seen susceptible mutations to reduce false positive resistance calls, the specificity of the Genoscholar PZA-TB II was 2.0% lower in silico than that of the catalogue mutations.

### Ethionamide

Molecular tests that identify *inhA* promoter mutations can diagnose resistance to ethionamide. For instance, the Xpert MTB/XDR test identified 47.3% of ethionamide resistance in silico in this dataset (Fig. 2). The catalogue includes mutations in an additional gene target, *ethA*, and these additional mutations result in a significantly higher sensitivity of 75.6%. However, the catalogue mutations do have a significantly lower specificity than Xpert MTB/XDR, which targets *inhA* promoter mutations only for ethionamide resistance detection.

### Fluoroquinolones

For both moxifloxacin and levofloxacin, all the tests performed as well as the catalogue mutations in terms of in silico sensitivity (Fig. 2). The resistance inferred interpretation of GenoType MTBDR*sl* version 2 identified the highest proportion of phenotypic resistance, with 84.7% sensitivity for detecting levofloxacin and 88.2% sensitivity for detecting moxifloxacin resistance in silico, but this was not significantly different from the other molecular diagnostics. In terms of specificity, for moxifloxacin, there was no significant difference between the catalogue or any of the molecular diagnostic tests. For levofloxacin, there was a higher specificity in silico using the resistance detected interpretation of the GenoType MTBDR*sl* v2 than the resistance inferred interpretation, and this was the only test with higher specificity than the catalogue. All the molecular diagnostic tests, for both levofloxacin and moxifloxacin resistance detection, had lower in silico sensitivity compared to the phenotypic DST than has been seen for the tests in clinical evaluation.

### Aminoglycosides

The catalogue mutations did not have significantly higher sensitivity than any of the molecular diagnostic tests for detecting amikacin resistance (Fig. 2). The resistance inferred interpretation of Genotype MTBDR*sl* version 2 had the highest sensitivity of any test at 77.3%, but this was not significantly higher than any of the other tests. Specificity was high (> = 99%) for the catalogue mutations and all tests evaluated in silico. We found that both resistance detected and resistance inferred interpretations of Genotype MTBDR*sl* v1 were the only results that had higher specificity than the catalogue.

Although capreomycin and kanamycin are no longer recommended for treatment of rifampicin-resistant or multi-drug resistant tuberculosis, they may still be in use in settings where a better alternative are not yet available and therefore we have included them in our analyses^57^. For capreomycin, the catalogue mutations, which include *tlyA* mutations, had significantly higher sensitivity in silico than all the molecular tests which only interrogate *rrs* and *eis* genes, and there was no significant difference in their specificities (Fig. 2). Of the molecular diagnostic tests, Genotype MTBDR*sl* version 2 identified the highest proportion of resistance, with 62.2% sensitivity in silico, however this was not significantly higher than any of the other molecular diagnostic tests.

For kanamycin, Xpert MTB/XDR and a resistance inferred interpretation of GenoType MTBDR*sl* version 2 had higher sensitivity than the other tests in silico, and no significant difference in sensitivity or specificity from the catalogue mutations (Fig. 2). The resistance inferred interpretation of GenoType MTBDR*sl* version 2 performed significantly better than the mutation only interpretation in terms of sensitivity, but for GenoType MTBDR*sl* version 1, where the *eis* promoter is not interrogated, both region and mutation interpretations performed equally. Specificity was > 98.0% for all the tests and the catalogue mutations and the tests with the lowest sensitivity had higher specificities than the catalogue mutations.

The molecular diagnostic tests for resistance to aminoglycosides had lower in silico sensitivity than that seen clinically, bar Xpert MTB/XDR, which had similar sensitivities to the clinical measurements for detecting amikacin and capreomycin resistance (Fig. 2). The differences between in silico and clinical sensitivities may not be significant as the clinical data has large associated confidence intervals due to small sample sizes (Table 3).

## Discussion

We found that for the drugs studied, the mutations detected by molecular diagnostic tests identify the majority of resistance, even in this diverse dataset, and performed comparably to the WHO mutation catalogue version 1^15^ for moxifloxacin, levofloxacin, amikacin and kanamycin and better than the catalogue for pyrazinamide resistance detection (Figs. 1, 2). However, the sequencing approach had higher sensitivity in silico than any of the PCR-based diagnostics for rifampicin, isoniazid, ethionamide and capreomycin (Figs. 1, 2).

### Molecular diagnostic tests perform similarly in silico compared to sequencing for identifying resistance to fluoroquinolones and two aminoglycosides

Specifically, for levofloxacin, moxifloxacin, amikacin and kanamycin, there was no significant difference between the in silico performance of the mutations covered by some molecular diagnostic tests and the catalogue (Fig. 2). This is an encouraging result, as areas of high TB burden may rely on molecular diagnostic tests as cheaper and more easily implementable alternatives to current sequencing approaches^58^ for certain drug resistance detection.

For pyrazinamide, the mutations identified by Genoscholar PZA II identified a significantly greater proportion of resistance than the catalogue (Fig. 1). Unlike with other drugs, there is no hotspot region of resistance mutations in *pncA,* and thus there are many rare mutations conferring pyrazinamide resistance across the gene and promoter region^59,60^, which the Genoscholar PZA II probes fully cover (Table 1). The WHO mutations catalogue however, is a first iteration, and included only mutations with sufficiently high confidence scores that were included as ‘associated with resistance’ or ‘associated with resistance – interim’ and therefore rare resistance conferring mutations in *pncA* may not have been included^15,24^. As more resistant samples are included in future iterations of the WHO mutations catalogue, it is likely that more rare mutations with ‘uncertain’ significance will be pushed over the threshold and be considered associated with resistance.

### The difference in the in silico performance of molecular tests was most pronounced for molecular tests that covered greater numbers of mutations

Despite differences in the mutations covered by molecular diagnostic tests, for moxifloxacin, levofloxacin, amikacin and capreomycin there was no significant difference in in silico sensitivity between any of the tests (Fig. 2). However, we found that increasing the number of mutations probed by molecular diagnostic tests can improve sensitivity for detecting resistance for the other drugs, and this is best exemplified by the data for rifampicin (Fig. 1). When using four specific mutation probes to detect resistance, as for resistance detection interpretations of Genoscholar NTM + MDRTB detection kit v2, GenoType MTBDR*plus* ver 2.0 and INNO LiPA MTB Rif, 80.1% sensitivity was achieved. After increasing the number of specific mutations detected to 13, 18 and 32 as for Truenat MTB/RIF, cobas MTB/RIF, FluoroType MTBDR VER 2.0 respectively, the sensitivity incrementally improved (Fig. 1). When the tests used any mutations within the *rpoB* RRDR to infer resistance, the sensitivity further increased, however there was a coincident decrease in specificity (Fig. 1). This is important to consider when using the tests in regions where non-resistance conferring mutations within the RRDR might be more prevalent and when using the WHO expert rule (any mutation in the RRDR is used as an indication of resistance in lieu of phenotypic DST results) to interpret NGS results^4,61^.

While molecular tests with the most comprehensive mutation set may seem like the optimal choice for detecting drug resistance, it is important to consider a range of other performance characteristics when evaluating a molecular diagnostic for clinical use. These include the test’s analytical sensitivity and specificity for tuberculosis detection (if it is being used as the primary diagnostic instead of reflex), ability to detect minor variants and reproducibility as well as factors such as cost and ease of sample preparation and processing^58^.

### The in silico performance of molecular diagnostic tests for pyrazinamide, fluoroquinolone and aminoglycoside resistance is lower than their clinical performance

For pyrazinamide, amikacin, capreomycin, kanamycin, levofloxacin and moxifloxacin, in silico sensitivities of the molecular tests were generally lower than what has been reported in the literature (Figs. 1, 2). Resistance to these drugs is less common than for isoniazid and rifampicin^1^, and therefore clinical resistant sample sizes are smaller and are likely to only contain the most prevalent resistance conferring mutations. However, the data used for our in silico analysis was drawn from a large sample set collected for the purpose of capturing rare phenotypes and diverse genetics for compiling the WHO mutations catalogue^15,24^, and as such is likely to have greater diversity than we would expect to see in clinical trials, particularly if trial samples are drawn from homogenous geographical regions.

### Sequencing has better in silico performance than molecular diagnostic tests for identifying phenotypic resistance to rifampicin, isoniazid, ethionamide and capreomycin and has additional benefits

Our analysis demonstrates that in silico sequencing-based DST performed better than in silico molecular diagnostic tests for rifampicin, isoniazid, ethionamide and capreomycin resistance detection (Figs. 1, 2). The advantage is that sequencing-based DST can be used to identify resistance across a broad genetic region allowing for resistance detection of drugs that have a more complicated genetic basis. For example, the Xpert MTB/XDR test, which detects *inhA* promoter mutations (important for resistance to both isoniazid and ethionamide^62,63^) but none of the ethionamide resistance conferring mutations occurring elsewhere in the genome, only identified 47.3% of the phenotypic resistance present in the data. The catalogue, which includes mutations in the *ethA* gene in addition to *inhA*, fared significantly better at 75.6% sensitivity, and this difference highlights the importance of probing multiple gene targets when they are implicated in phenotypic resistance.

The higher sensitivity for rifampicin resistance detection of the WHO catalogue compared to PCR-based diagnostics in silico, may be due to the presence of resistance conferring mutations outside of the RRDR, such as *rpoB* I491F which are present in the catalogue but not detected by the molecular diagnostics (Table 1)^9,15^. The rifampicin resistance associated *rpoB* I491F mutation is particularly prevalent in Southern Africa, meaning that molecular based diagnostic performance will vary based the prevalence of specific mutations in various geographical regions, whereas sequencing would include an exhaustive mutation set, reducing the impact of variations in regional prevalence.

There is a range of other benefits sequencing can provide in comparison to molecular diagnostic tests. Increased uptake and use of NGS, and especially WGS, will also allow new mutations to be identified as they emerge and will enable further associations with resistance to be made. As new resistance-associated mutations are added to the catalogue, molecular diagnostic tests will not be able to adapt their landscape for resistance detection easily or quickly, whereas sequencing would only need a bioinformatics update and, in the case of tNGS platforms, the addition of a new primers if additional genes are needed. An additional benefit of sequencing is the ability to distinguish between mutations causing higher and lower levels of resistance as patients could benefit from different treatment regimens and dosages^64^, this is straightforward with sequencing but is not possible with molecular tests that infer resistance. It is important to note that distinguishing between different levels of resistance is also possible for molecular diagnostic tests that use specific mutation probes^65^ and with the Xpert MTB/XDR test for isoniazid and fluoroquinolone resistance^42^. Finally, while heteroresistance was not considered in this study, mixed populations can be detected and quantified using tNGS^66^ and WGS^67^, however we note that tNGS may be the preferable option as the culturing step required for WGS could prevent the detection of mixed infections^68^.

### Comparison of in silico performance of mutations identified by the WHO 2021 catalogue and molecular diagnostic tests to phenotypic DST

Overall, mutations identified by the catalogue and available molecular diagnostic tests had higher sensitivity for detecting rifampicin resistance than other antitubercular drugs; several tests identified over 90% of rifampicin resistant isolates in the dataset. This illustrates how rifampicin resistance conferring mutations have been well characterised^69–72^ and rapid molecular tests are thus also well suited for identification of resistance.

For all drugs bar rifampicin and isoniazid, both molecular diagnostic test and catalogue sensitivity in silico were below 90%, the target product profile for the minimal criteria to identify resistance at peripheral centres^58^. For pyrazinamide, ethionamide, amikacin and capreomycin, the in silico sensitivity of the catalogue and all molecular diagnostic tests particularly low, less than 80%. This illustrates the need for further work identifying mutations associated with resistance to these drugs. Overall, the sensitivity of the catalogue and all molecular diagnostic tests for fluoroquinolones is also low (Fig. 2), despite resistance being well attributed to key regions in the *gyrA* and *gyrB* genes^73^. This could be driven by heterozygous calls at less than 90% fraction read support that were not identified in the isolates used in this analysis. Significant fluoroquinolone heteroresistance has been observed in *M. tuberculosis*^74,75^, yet clear thresholds for clinically significant heteroresistance have not been set. Our understanding of the genetic determinants of resistance is far from exhaustive, and complex genetic resistance signatures involving increased efflux, metabolic changes and membrane permeability have yet to be characterized, thus limiting the sensitivity of diagnostics.

The specificity of both the catalogue and the tests was generally high in silico, and in line with the WHO optimal requirements at over 98%^58^, for all drugs except for moxifloxacin, ethionamide and pyrazinamide (Figs. 1, 2). We attribute the low in silico specificity for moxifloxacin resistance detection to the use of historical data with critical concentrations that are no longer recommended for defining resistance^64^.

### Limitations

Because no other comparably large dataset was available the dataset used for the in silico analysis was the same dataset that used to create the catalogue and therefore the in silico performance of the catalogue may be an overestimate compared to real world performance, a limitation in our study. Also, to ensure a large sample set, the phenotypic data used was collected using different breakpoints. Continual updates to critical concentrations highlight reliability problems with the phenotypic DST standard^61,64^. Furthermore, several isolates containing common and well characterised resistance conferring mutations, such as *katG* S315T and *rpoB* S450L, were reported as phenotypically susceptible to isoniazid and rifampicin. Possible explanations include labelling errors during phenotypic testing^15,76^, or incorrect phenotypic DST. Despite the limitations associated with phenotypic DST, it will continue to be required as a first step to identify new resistance mechanisms and associations as these cannot be identified using sequencing alone.

## Conclusion

In conclusion, NGS has many benefits in comparison to PCR-based molecular diagnostic tests. As NGS is not yet feasible as a universal solution to fit all DST needs, it is encouraging to find that the mutations probed by molecular PCR-based diagnostics capture a high proportion of drug resistance in this diverse dataset. We hope that this dataset, and the presented in silico method, could help quickly evaluate lists of mutations for new or revised drug resistance assays. Although there are many advantages and limitations to consider for any diagnostic test, it is clear that a variety of diagnostic tools, including both targeted molecular and NGS based solutions, and phenotypic DST to identify resistant isolates and associated resistance mechanisms, will be vital to improve detection of antibiotic resistance and ultimately eliminate TB.

## Supplementary Information

Supplementary Tables.

## Data Availability

All data used and generated during this study are included in this published article (and its Supplementary Information files). Data analyzed in this study were a re-analysis of existing data, which are openly available at locations cited in the reference section, but the matched genetic and phenotypic data for all isolates, including European Nucleotide Archive accession numbers for all the raw sequencing data, are presented in Table S4.
